# New Trends in Biopolymer-Based Membranes for Pervaporation

**DOI:** 10.3390/molecules24193584

**Published:** 2019-10-05

**Authors:** Roberto Castro-Muñoz, José González-Valdez

**Affiliations:** 1 Tecnologico de Monterrey, Campus Toluca. Avenida Eduardo Monroy Cárdenas 2000 San Antonio Buenavista, 50110 Toluca de Lerdo, Mexico; 2 Tecnologico de Monterrey, School of Engineering and Science, Av. Eugenio Garza Sada 2501, Monterrey, N.L. 64849, Mexico

**Keywords:** pervaporation, mixed-matrix membranes, polar compounds, non-polar compounds, biopolymers

## Abstract

Biopolymers are currently the most convenient alternative for replacing chemically synthetized polymers in membrane preparation. To date, several biopolymers have been proposed for such purpose, including the ones derived from animal (e.g., polybutylene succinate, polylactic acid, polyhydroxyalcanoates), vegetable sources (e.g., starch, cellulose-based polymers, alginate, polyisoprene), bacterial fermentation products (e.g., collagen, chitin, chitosan) and specific production processes (e.g., sericin). Particularly, these biopolymer-based membranes have been implemented into pervaporation (PV) technology, which assists in the selective separation of azeotropic water-organic, organic-water, organic-organic mixtures, and specific separations of chemical reactions. Thereby, the aim of the present review is to present the current state-of-the-art regarding the different concepts on preparing membranes for PV. Particular attention is paid to the most relevant insights in the field, highlighting the followed strategies by authors for such successful approaches. Finally, by reviewing the ongoing development works, the concluding remarks and future trends are addressed.

## 1. Introduction

Biopolymers are commonly obtained from different sources, including animal (e.g., polybutylene succinate, polylactic acid, polyhydroxyalcanoates), vegetable sources (e.g., starch, cellulose-based polymers, alginate, polyisoprene), bacterial fermentation products (e.g., collagen, chitin, chitosan), and specific production process (e.g., sericin which is a by-product of the silk processing process). These biomaterials are currently recognized as potential materials for replacing chemically synthetized polymers according to the current green environment-based initiatives and regulations [1]. To date, such biopolymers have been widely proposed for several applications, such as drug delivery systems, biomedicine, hydrogels, water treatment, food packaging, and membranes, to mention just a few of them [2]. When dealing with membrane preparation, biopolymer-based membranes have been applied into several membrane technologies, such as microfiltration, ultrafiltration, pervaporation, gas separation, and membrane for tissue engineering. Particularly, pervaporation (PV) is recognized as one of the most selective membrane-based technologies, which has been successfully applied for the separation of several types of close-boiling azeotropic mixtures, including organic-organic, water-organic, and organic-water mixtures [3,4,5]. In this membrane technique, synthetic polymers (e.g., polyvinyl alcohol (PVA), polydimethylsiloxane, polyimides, poly(1-(trimethylsilyl)-1-propyne), poly(octylmethylsiloxane)) have been the most used materials in the preparation of membranes [6,7,8,9]. Regarding this, Table 1 reports some examples of the most common azeotropic mixtures separated by means of synthetic PV membranes. It can be seen that hydrophilic polymers have been the most used for their capability of removing polar compounds.

The separation performance of PV membranes depends on the intrinsic properties of the polymers (e.g., hydrophilicity, hydrophobicity, free volume) [21,22]. Moreover, the distribution of the dissolved molecules in the polymeric membrane depends on the affinity between the penetrant molecules and the membrane. This affinity can be interpreted by considering Hansen′s solubility parameters between both phases. Hansen solubility parameters generally denote the dispersive (δd), polar (δ_p_), and hydrogen bonding (δ_h_) contributions, while the total solubility (δ_T_) is the contribution of those three imposing concepts [23]. In theory, closer values of the solubility parameters express higher compatibility and therefore solubility of a polymer–solvent pair [24]. Unfortunately, such synthetic polymers present trade-off limitations according to their properties. For example, highly selective polymers do not display high permeation rates, while highly permeable polymers are not selective enough. Today, biopolymers are gaining researchers’ attention within PV applications due to their intrinsic features which have provided relevant insights when comparing their separation performance with synthetic polymers. Thereby, the aim of this review article is to provide the current developments in works related to the use of different biopolymer-based membranes in PV separations, providing the new trends and future developments in the field. Moreover, the main drawbacks of such biopolymers are addressed, giving potential suggestions for new researchers in the field.

## 2. Biopolymers: The Promising Materials in Membrane Preparation for Pervaporation Operations

**Figure 1:**
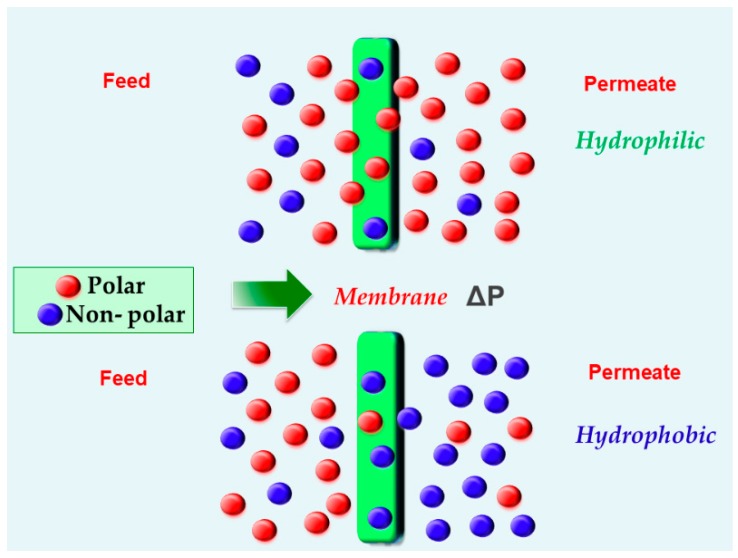
Graphical depiction of the preferential transport of hydrophilic and hydrophobic membranes.

As previously mentioned, PV is a highly selective membrane technology which is able to separate close-boiling azeotropic mixtures by partial vaporization of the components in the solution. This selective separation is carried out by using a dense non-porous membrane. In principle, the azeotropic feed mixture is in direct contact with the “selective” side of the membrane, while the permeate, enriched by the species with higher affinity for the membrane (hydrophilic or hydrophobic types) (see Figure 1), is collected on the other side. The use of hydrophilic membranes implies the preferential transport of polar molecules over non-polar molecules. Hydrophilic polymers preferentially allow the water solubility (and some other highly polar molecules) across the membrane through hydrogen-bonding interactions. Importantly, these types of membranes are commonly affected by the swelling phenomenon, in which the membranes are susceptible to water or polar organic molecules [25]. This phenomenon has been identified as the main drawback of hydrophilic PV membranes. Swelling is denoted as the reduction in the interaction between segments of the polymer chains caused by polar compounds, and thus, an increase in segmental mobility and free volume. As a consequence, the swelling increases the permeation rates (i.e., permeate flux), while a decrease in selectivity is observed due to the plasticization effect [26]. On the other hand, hydrophobic polymers allow the transport of less polar (or nonpolar) molecules contained in the azeotropic mixtures [27].

The transport of the permeating species occurs thanks to the driving-force applied: (i) vacuum pressure or (ii) sweeping gas (like nitrogen) and (iii) temperature. The transport mechanism through dense polymeric membranes has been widely explained by the well-known solution-diffusion model [28,29], which describes the mass transfer through a PV membrane as follows: (i) adsorption of the target molecules from the bulk mixture to the “selective” side of the membrane on the basis of their chemical affinity, afterwards; (ii) a diffusion of the molecules through the membrane takes places as a result of the concentration gradient; and finally, (iii) a subsequent desorption of the molecules at the permeate side of the membrane occurs [30]. The mass transport is governed by the chemical potential (μi) gradient, the physicochemical properties of the permeating molecules, (*i*) and their concentration in the feed and permeate sides. Finally, the permeability (P) depends on the diffusivity (D) and solubility (S) of the target molecules [28,29]. In general, the performance of a PV process is commonly evaluated in terms of permeate flux (J) and separation factor (β) [31]. For instance, the permeate flux (J_A_) of compound A (denoting the faster permeating molecules) can be determined by Equation (1), where m_A_ is the mass of molecule A transported through specific membrane area (A_m_) in a specific operating time [32,33]:

(1) $$J_{A}\;=\;\frac{m_{A}}{A_{m}\;\cdot \;t}$$

β factor provides the relationship between the concentration of compound A and B in permeate and retentate streams, as shown by Equation (2):

(2) $$\beta_{A}\;=\;\frac{{\left(\frac{C_{A}}{C_{B}}\right)}_{permeate}}{{\left(\frac{C_{A}}{C_{B}}\right)}_{feed}}$$

PV has emerged as a potential candidate for replacing conventional distillation operations [34]. This membrane technique provides less productivities in terms of flux than PV. However, to meet high purification degrees reached by PV, conventional distillation must involve the implementation of at least a couple of distillation columns [35,36], which definitely influences the energy demand and its overall consumption in the process. This energetic cost strongly makes an impact on the economic evaluation of the procedure [37]. Importantly, when using PV technology, the production demand can be archived by handling operating parameters (e.g., driving force, membrane area, feed concentration, feed temperature) [35]. To date, synthetic polymeric-based membranes have been widely applied in PV technology over the last decades [38,39]. However, biopolymer-based membranes have recently emerged as potential materials in replacing such synthetic polymers, achieving the appearance of novel and effective membrane fabrication materials that look for more environmentally friendly processes. Moreover, the separation features of biopolymers in comparison with conventional polymers have also promoted their application in PV separations, in which biopolymers have provided superior performances. Table 2 reports the most recent advances on the use of biopolymer-based membranes in the field. 

### 2.1. Chitosan-Based Membranes

**Figure 2:**
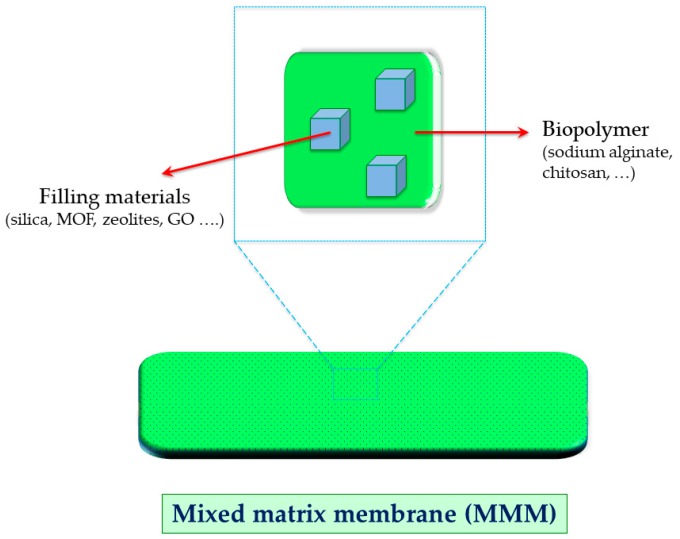
Representative scheme of a biopolymer-based mixed matrix membrane.

The current need for improving the performance of biopolymer materials in PV has brought the implementation of mixed matrix membranes (MMMs) which implies the embedding of inorganic materials into the biopolymer-based membrane [40,41]. The limitation in separation performance has promoted to look for novel materials which may enhance different membrane features [42,43]. Figure 2 depicts a general scheme of MMMs using biopolymers. To date, Chitosan (CS) is likely the most explored biopolymer in the fabrication of MMMs. Such biopolymer is derived from the deacetylation reaction of chitin (poly(β-(1-4)-*N*-acetyl-d-glucosamine). This primary material is obtained by an extraction procedure of the outer shell of crustaceans. Once its deacetylation degree reaches a value of around 50%, CS is obtained. When dealing with MMMs, ultrathin chitosan (CS)–titania hybrid membranes for ethanol dehydration have been proposed by Zhao et al. [44]. The embedding of the inorganic metal (titania) has allowed the simultaneous enhancement of hydrophilicity and membrane swelling resistance. These enhancements were mainly associated with the presence of numerous carboxyl groups and the stable hybrid structure. The tested operations presented the best performance in the MMMs containing 14 wt.% of titania, displaying a permeate flux and separation factor (β) of 1.40 kg m^−2^ h^−1^ and 730, respectively. In practice, MMMs kept the initial permeation fluxes of the pristine CS (1.50 kg m^−2^ h^−1^). Nonetheless, the authors greatly managed to concurrently enhance selectivity.

More recently, Gao et al. [54] prepared CS MMMs containing 10 wt.% potassium montmorillonite (K+MMT), which presented a permeation rate in the range of 1.50 kg m^−2^ h^−1^, similar to that of pristine CS, in acetone dehydration experiments. However, the K + MMT-doped MMMs provided a higher β value of about 2200, which was around eight times higher than the pristine CS (β value of 249). The authors described that the ionized water channel constructed by the MMT intergallery within the hybrid membrane may have greatly contributed to the β enhancement. In addition, the increase could also be attributed to: (i) the fact that MMT embedding increases the surface energy of polar molecules, therefore, directly promoting the preferential transport of water, or (ii) the creation of additional diffusional pathways for water selective transport [54]. Using other types of filling materials, Kang et al. [49] incorporated ZIF-7 in order to synthesize high-performance pervaporation MMMs based on ZIF-7-CS membranes. Surprisingly, the separation efficiency of MMMs, containing only 5 wt.% ZIF-7, was 19 times higher than that of the pristine CS membranes when dehydrating ethanol. Such suppressive enhancement on the β value was related to the rigidified polymer chain in the MMMs. Moreover, changes in the d-spacing of the ZIF-7 crystals were also documented, caused by the interpenetration of CS polymer into the ZIF-7 frameworks, which produced polymer chain rigidification. This rigidification phenomenon allowed a decrease in both flux and β. In general, the water molecules (with a kinetic diameter of 2.96 Å) cannot be transported through the ZIF-7, which possess a pore size of 2.9 Å). This particular feature of the metal-organic framework (MOF) may have also contributed to the decrease in permeation fluxes, e.g., from 0.60 to 0.32 kg m^−2^ h^−1^. Moreover, the incorporation of a cross-linker, like glutaraldehyde (GA), also produced enhancements in selectivity. Very recently, Li et al. [55] embedded into CS another hydrophilic MOF-801, which revealed a superior water adsorption capacity. These MOF-801 MMMs presented an optimal separation efficiency for the separation of water–ethanol mixtures containing only 4.8 wt.% filler loading, being the permeate flux and separation factor of 1.100 kg m^−2^ h^−1^ and 2156, respectively. Particularly, these CS-based MMMs have exhibited higher performances than other MMMs based on synthetic polymers aiming at ethanol dehydration (see Table 3). It is worth mentioning that these particular MMMs (i.e., MOF-801 MMMs) can even compete with commercially consolidated polymers used in industry for dehydration of organics, like PVA, considering that researchers have reported permeation fluxes in the range of 0.080–0.137 kg m^−2^ h^−1^ for such PVA MMMs. 

The incorporation of inorganic materials has provided enhancements to CS separation performance. However, the incorporation of the inorganic phases does not guarantee the improvement of both properties (i.e., permeation and selectivity), there are still some issues, like interaction lacking between filler-CS, that is forcing today’s research community to implement new strategies, including chemical modification of the inorganic filling materials, and thus, obtaining superior performing membranes. In this context, several inorganic nanomaterials have been chemically modified and therefore incorporated into CS-based membranes, such as graphene [69], graphene oxide [65], silicas [70], carbon molecular sieves [64], multiwalled carbon nanotubes [63], among others. 

In this context, Ag^+^ grafted nanotubes were filled in CS and afterward deposited onto a polysulfone support [69]. The fluxes of those thin-film membranes increased concurrently with the selectivity at a specific filler loading (0.5–1.5 wt.%), revealing that an improvement in the filler-CS compatibility can be reached by chemical grafting. The membranes possessed a high permeation flux ~0.44 kg m^−2^ h^−1^ towards the dehydration of 50 wt.% benzene in feed (at 60 °C). 

Nanocomposite membranes based on functionalized graphene sheets (2.5 wt.%) and CS provided high selectivity values for water–isopropanol and water–ethanol mixtures of 7781 and 1093, respectively [69]. The sulfonation of carbon molecular sieves (CMS) has been performed to fabricate hybrid membranes [64]. Herein, the purpose of sulfonation was to change the CMS hydrophobic nature to hydrophilic. Such sulfonated CMS were later incorporated into CS. Thanks to the ordered mesoporous structure of the material, the authors were able to prepare membranes which presented higher water transport pathways, giving a positive feature together with the hydrophilicity increment. Especially, the membranes containing 2 wt.% modified CMS displayed an optimal separation performance with a β of 832 and a permeate flux of 1.81 kg m^−2^ h^−1^ in water–acetone separation. In general, it can be concluded that the chemical modification of the filler allowed the increase by two-fold of the β factor and 1.67 times for the permeation flux when compared to the pure CS membranes. 

### 2.2. Sodium Alginate-Based Membranes

**Figure 3:**
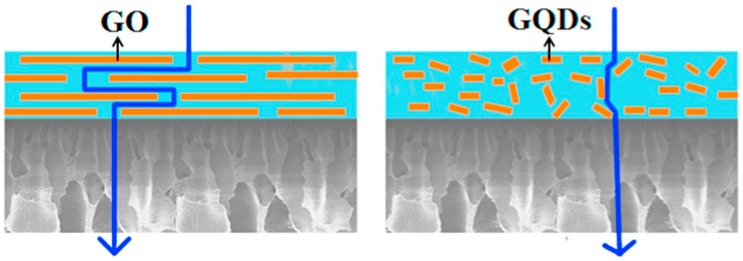
Schematic depiction of water permeation in nanocomposite PEG@POSS-SA membrane. Taken from Wang et al. [48].

Sodium alginate (SA) has been another potential biopolymer used in membrane preparation for PV. This biopolymer is a sodium salt form of alginic acid and gum mainly extracted from the cell walls of brown algae. Recently, graphene oxide (GO) quantum dots were incorporated into nanocomposite membranes, displaying high total permeate flux values of 2.4 kg m^−2^ h^−1^ for pervaporative ethanol dehydration [48], which represented a 60% increment in that of pure alginate membranes. Regarding the separation factor, such membranes displayed a value of 1152. Finally, the authors claimed that the embedding of the incorporation of the GO dots improved the water permeation across the biopolymer membrane, which was properly related to the filler properties and its features. In Wang’s study, the description of the possible water permeation for GO dots was compared to the pristine GO, as Figure 3 illustrates:

Basically, it is believed that the different permeation paths in the various fillers embedded in nanocomposite membranes could be the result of different transport resistances. For example, in GO–composite membranes, the GO sheets with high aspect ratios and lateral sizes tend to disperse horizontally in the polymer matrix [10,71]. In this sense, the transport pathways are becoming longer and more tortuous, provoking the permeating molecules to have a zig-zag movement through the close-packed GO channels [72]. While in the nanosized GO dots, unlike the barrier effect of GO, the dot flakes can create a relatively shorter way with lower transfer resistance for water molecules, which definitely leads to a rise in the permeation rate. Thereby, it is clear that the filler distribution over the membrane can strongly influence its separation performance [73], in which the preparation protocols used in the membrane fabrication are also playing a fundamental role [74,75,76].

**Figure 4:**
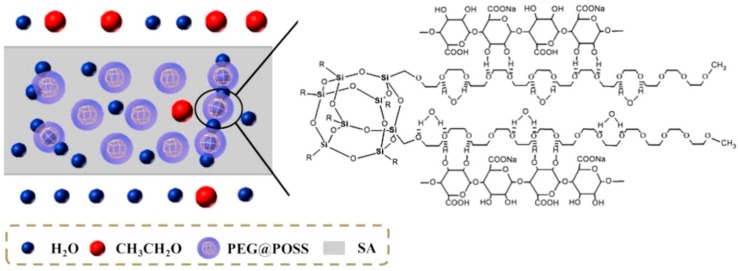
Schematic depiction of water permeation in nanocomposite PEG@POSS-SA membrane. Taken from Wang et al. [46].

Wang et al. [46] used poly(ethylene glycol) variants as the main matrix for the preparation of MMMs aimed at ethanol dehydration. In this case study, the author synthetized poly(ethylene glycol)-functionalized polyoctahedral oligomeric silsesquioxanes (PEG@POSS) nanoparticles, which were later incorporated into SA. The functionalization of the POSS provided a large quantity of hydrophilic PEG side chains, which enhanced the hydrophilicity of the membrane. This feature offered massive water binding sites in the membranes (see Figure 4). 

This smart design of the nanostructured material allowed the obtention of greater separation performance. For example, MMMs loaded with 30 wt.% filler exhibited a maximum permeation flux of about 2.5 kg m^−2^ h^−1^ and separation efficiency of 1077. When comparing such separation performances, it seems to be like these specific designed membranes may offer even higher permeation rates compared to CS MMMs. Interestingly, the superior performance was caused by the fact that an ethylene glycol (EG) unit can bind water molecules through hydrogen bonds and form a hydration layer along the PEG chain. Herein, PEG (n = 13.3) was used to modify POSS (PEG(n)@POSS (n = 13.3), meaning that 104 water molecules could gather around the PEG chains [46]. Moreover, other important phenomena may play an important role. For instance, a decrease in crystallinity together with the increase of fractional free volume can provide water molecules with a fast transport pathway in the free volume cavities between the biopolymer chains, which is actually leading to the enhancement of permeation rates. In addition to this, the improvement of the hydrophilicity of the nanocomposite membranes after the embedding of PEG@POSS would preferentially adsorb water molecules and concurrently repel ethanol molecules.

Highly water-selective hybrid membranes, displaying superior water–ethanol pervaporative separation performance, were developed by incorporating g-C_3_N_4_ nanosheets (CNs) into SA [52]. The nanostructured material CNs were synthesized by thermal oxidation “etching” and ultrasound exfoliation methods. By incorporating 3 wt.% CNs, the membranes presented a permeation flux up to 2.4 kg m^−2^ h^−1^ while the β factor was around 1653 (i.e., 76 °C, 10 wt.% water concentration). When comparing these results with the ones reported by Wang et al. [46], the permeation fluxes were found in the same range, while the selective properties towards water of the CNs-SA membranes have been higher. Moreover, the separation performance of these membranes was notably stable over long operating times (e.g., 168 h). 

It is likely that the main task of biopolymer membranes is dealing with the separation of common azeotropic mixtures (mainly dehydration of organics). However, these emerging membrane materials have also been used for the optimization of specific reactions, such as esterification, acetalization, or etherification [33,77,78]. These reactions comprise the production of organics, like esters, acetals and some other valuable chemicals. Crucially, such processes are well recognized as equilibrium-limited liquid reactions. As reactions occur, water is produced as a byproduct, representing an issue because of the simultaneous hydrolysis of the produced molecules up to a point at which thermodynamic equilibrium is achieved. In order to overcome such an equilibrium and therefore enhance the conversion efficiency, the byproduct removal (i.e., water) has been the main strategy. Thereby, pervaporation membrane reactors (PVMR) are a latent concept which implies a hybrid process involving PV and a chemical reactor. Properly, the role of the PV technology is to remove out the byproduct once the reaction takes place. For this purpose, hydrophilic membranes with high affinity towards water are required. In the light of PVMR, a dual-function catalytic composite membrane reactor composed by a sulfonated polyvinyl alcohol (SPVA) casting solution onto a PVA- SA membrane has been developed by Bo et al. [79]. Such design resulted in the conversion of over 96% in the production of ethyl acetate (esterification of ethanol and acetic acid). Importantly, the conversion of reactants was enhanced by 24% compared to a batch reactor. In terms of stability, the PVMR showed that the acid conversion efficiency could still reach up to 78% in the fifth successive run.

### 2.3. Other Biopolymer Membranes

Some other types of biomaterials have also been presented as possible membrane materials for PV purposes. One of them has been polylactic acid (PLA). PLA is a commercially available polymer obtained from lactic acid. Typically, PLA is synthetized by polycondensation of hydroxyl acid or by ring-opening polymerization of cyclic lactide monomers [1]. This polymer has been recently evaluated for its ability in separating methanol (MeOH)-methyl tert-butyl ether (MTBE) azeotropic mixtures [60]. The separation performance in terms of permeation and selectivity was about 0.090 kg m^−2^ h^−1^ and 75, respectively. In this study, the polymer was basically tested in its pristine form; regardless of this, it provides important selective properties. 

Some polysaccharides recovered from microbial fermentation broths have also been explored in the field [80,81]. Meireles et al. [81] evaluated the performance in the dehydration process of exopolysaccharide, FucoPol, produced by Enterobacter A47. The polysaccharide-based membranes displayed comparable selective properties (β = 566, in ethanol dehydration) compared to conventional synthetic polymers (like PVA). Nevertheless, when compared to other biopolymers, such as CS or SA, both properties, permeability and selectivity, are still far from comparable.

A highly hydrophilic macromolecular biocompound, like sericin, was used for its blending with PVA, attempting ethanol dehydration [82]. Such protein comprises 18 amino acids that possess mostly strong polar sides (e.g., hydroxyl, carbonyl and amino). Such polarity provides the material with the potentiality to improve the hydrophilic nature of hydrophilic materials. In PV testing, the blended membranes showed high selectivity towards water in comparison with ethanol, achieving a β factor of 145–172 with a total flux between 0.05 and 0.10 kg m^−2^ h^−1^ measured at 8.5 wt% water in the feed (at 50–70 °C). Polyhydroxybutyrate (PHB) is another bio-derived and biodegradable polyester. PHB is the most well-known polymer of the PHA family. PHB is actually the simplest and most common PHA produced by microorganisms. It is recognized to be produced by several bacteria, including the genres Alcaligenes, Azobacter, Bacillus, and Pseudomonas [1,83]. Such bio-polyester was employed for the preparation of dense PV membranes looking for the separation of methanol–MTBE and methanol–water mixtures [84,85]. Such PHB membranes, in both separations, presented acceptable structural stability to swelling. In general, the membranes displayed high flux although with moderate selectivity values. Especially, in methanol–MTBE separation, the acrylic plasma-polymerized PHB membranes revealed higher selectivity values than the ones reported for pure PHB membranes, reaching values up to 18.6 (at 40 °C, 20 wt.% of methanol). Whereas in the case of methanol–water separation, a β factor in the range of 3.5–3.8 and total fluxes between 0.13 and 1.4 kg m^−2^ h^−1^ were obtained when the operating temperature was increased from 30 to 50 °C. To date, there is no report about using these latest bio-derived materials in the MMM concept, which, according to their promising insights in polar separations, may be explored by the research community.

## 3. Concluding Remarks

Over the course of this review, biopolymers (e.g., chitosan, sodium alginate, and some other biomaterials) were presented as examples for continuous matrixes in membrane preparation for the purpose of azeotropic separations by means of pervaporation approaches. The success of these materials deals with their high affinity towards polar compounds (e.g., water), good film-forming features, and the presence of plenty functional groups that provide the possibility of chemical modification and blending with other materials (polymers). Their mechanical stability has nevertheless been attempted to be enhanced by coating onto porous supports and crosslinking with different substances (i.e., glutaraldehyde and sulfuric acid). Additionally, the physical merging as MMMs with inorganic nanomaterials (e.g., MOFs, zeolites, molecular sieves, nanotubes, graphene oxide, among others) has corroborated the enhancement of mechanical properties by restricting the polymer chain mobility which provides better stability against swelling in long-term separation operations especially in the presence of polar molecules.

On the basis of poor performance either by efficiency or permeation because of the poor filler-biopolymer compatibility, the current trend in mixed matrix membrane preparation is strongly focused on the chemical functionalization of fillers and the improvement of their interfacial adhesion into the polymer matrixes. Such chemical modification also allows the use of minimal filler loadings. Critically, the incorporation of large filler amounts tends to provide more highly permeable membranes with the offset of compromising other selective properties. This is due to the fact that new non-selective pathways facilitate the transport of larger molecules. At this point, the smart selection of suitable inorganic materials, like the chemically modified ones, is important to reach the target enhancement that could demand a low filler loading. Importantly, the use of low filler loadings will promote the reduction of the cost of membrane fabrication. Furthermore, lower filler loadings may diminish some other issues in MMM preparation. For instance, particle agglomeration and consequently non-selective voids have been identified with this issue. Thereby, a good adhesion may result in a featured interface between the inorganic particles and the biopolymer matrix which could determine the right path to be passed through by certain molecules over others. This feature will allow the selective transport of certain molecules expecting enhanced selectivity; or in some specific cases, it may also result in raised permeability by decreasing the pathlength of the permeating molecules. In this way, specific biopolymer mixed matrix membranes (e.g., PEG@POSS-SA, CNs-SA, MOF-801-CS) have displayed greater permeation rates and notably excellent separation properties. It is important to highlight that the low permeation rates of the PV technology have also been identified as one of the main bottlenecks of this technique, in which biopolymer membranes have shown to overcome such issue.

Finally, the current insights on using biopolymers for PV membranes will encourage the use of those materials in the coming future, in which their role as emerging environmentally friendly materials will also support their usage according to today’s green environment-based initiatives and regulations. Furthermore, the design and implementation of “green” technologies, like PV, according to the “Twelve Principles of Green Chemistry”, will promote the establishment of these materials in larger operations.

## Figures and Tables

**Table 1:** Azeotropic separations using synthetic PV membranes.

Azeotropic Mixture	Type of Separation	Membrane Used	Membrane Type	Reference
Water/ethanol	Water-organic	Polyvinyl alcohol (PVA)	Hydrophilic	[10,11]
Water/isopropanol	Water-organic	Polyvinyl alcohol (PVA)	Hydrophilic	[12]
Ethanol/water	Organic-water	Polyacrylonitrile (PAN)	Hydrophilic	[13]
Ethanol/water	Organic-water	Polyvinylidene fluoride (PVDF)	Hydrophobic	[14]
Ethanol/water	Organic-water	Polydimethylsiloxane (PDMS)	Hydrophobic	[15,16]
Butanol/water	Organic-water	Polyoctylmethyl siloxane (POMS)	Hydrophobic	[17]
Toluene/methanol	Organic-organic	Poly(styrene-co-butadiene) rubber	Hydrophobic	[18]
Toluene/n-heptane	Organic-organic	Polyvinyl alcohol (PVA)	Hydrophilic	[19]
Methanol/MTBE	Organic-organic	Polyimide	Hydrophilic	[20]
Methanol/MTBE	Organic-organic	Polyamide-6	Hydrophilic	[7]

**Table 2:** Ongoing development works using biopolymer-based membranes for PV.

Polymer Matrix:	Filler:	PV Separation:	Operating Conditions:	Performance Unfilled Membrane	Performance Filled Membrane	Reference:
CS/PVP	Silica	MeOH-EG	60 °C, 6 wt.% MeOH, 10 mbar	J: 0.15 kg/m^2^ h β:500	At 28.4 wt.% loading: J: 0.06 kg/m^2^ h β: 6000	[45]
SA	PEG-POSS	Water-EtOH	77 °C, 10 wt.% water, 3 mbar	J: 1.8 kg/m^2^ h β:900	At 30 wt.% loading: J: 2.5 kg/m^2^ h β: 1077	[46]
CS/PVP	BTEE	MeOH-EG	60 °C, 6 wt.% MeOH, 10 mbar	J: 0.04 kg/m^2^ h β:500	At 10.4 wt.% loading: J: 0.05 kg/m^2^ h β:6129	[47]
CS	Titania	Water-EtOH	77 °C, 10 wt.% water, 30 mbar	J: 1.500 kg/m^2^ h β:200	At 14 wt.% loading: J: 1.403 kg/m^2^ h β: 730	[44]
SA	GO dots	Water-EtOH	76 °C, 10 wt.% water, 0 mbar	J: 1.500 kg/m^2^ h β:500	At 2 wt.% loading: J: 2.4 kg/m^2^ h β: 1152	[48]
CS	ZIF-7	Water-EtOH	25 °C, 10 wt.% water	J: 0.600 kg/m^2^ h β:148	At 5 wt.% loading: J: 0.322 kg/m^2^ h β:2812	[49]
CS	ETS-10	Water-EtOH	50 °C, 15 wt.% water, 2 mbar	J: 0.450 kg/m^2^ h β:47	At 5 wt.% loading: J: 0.550 kg/m^2^ h β:30	[50]
CS	TEOS	Water-EtOH	30 °C, 50 wt.% water	-	At 10 wt.% loading: J: 0.720 kg/m^2^ h β:450	[51]
SA	g-C_3_N_4_ nanosheets	Water-EtOH	76 °C, 10 wt.% water, 3 mbar	J: 1.500 kg/m^2^ h β:500	At 3 wt.% loading: J: 2.4 kg/m^2^ h β:1653	[52]
CS	ZIF-8	Water-IPA	25 °C, 15 wt.% water, 0.05 mbar	J: 0.325 kg/m^2^ h β:800	At 2.5 wt.% loading: J: 0.280 kg/m^2^ h β:800	[53]
CS	K^+^MMT	Water-AC	50 °C, 5 wt.% water, 0.03 mbar	J: 1.5 kg/m^2^ h β:250	At 10 wt.% loading: J: 1.7 kg/m^2^ h β:2200	[54]
CS	MOF-801	Water-EtOH	70 °C, 10 wt.% water, 3 mbar	J: 1.00 kg/m^2^ h β:700	At 4.8 wt.% loading: J: 1.100 kg/m^2^ h β:2156	[55]
CS	SiO_2_ xerogel	Water-BuOH	25 °C, 10 wt.% water	J: 0.400 kg/m^2^ h β:500	At 0.25 wt.% loading: J: 0.500 kg/m^2^ h β:1900	[56]
CS	NaY	Water-IPA	30 °C, 10 wt.% water, 10 mbar	J: 0.05 kg/m^2^ h β:2000	At 40 wt.% loading: J: 0.113 kg/m^2^ h β:11,000	[57]
SA	MoS_2_	Water-EtOH	77 °C, 10 wt.% water, 1 mbar	J: 1.2 kg/m^2^ h β:650	At 2 wt.% loading: J: 1.83 kg/m^2^ h β:1229	[58]
CS	TGDMP	Water-IPA	30 °C, 10 wt.% water, 10 mbar	J: 0.35 kg/m^2^ h β:600	At 1.2 wt.% loading: J: 0.737 kg/m^2^ h β:1050	[59]
PLA	-	MeOH-MTBE	40 °C, 14.3 wt.% MeOH, 6.1 mbar	J: 0.090 kg/m^2^ h β:75	-	[60]
CS	MXene	Water-DMC	50 °C, 2 wt.% water, 2 mbar	J: 1.0 kg/m^2^ h β:400	At 3.0 wt.% loading: J: 1.4 kg/m^2^ h β:900	[61]
CS	Al-MOF	Water-EtOH	25 °C, 10wt.% water	J: 0.383 kg/m^2^ h β:240	At 5 wt.% loading: J: 0.458 kg/m^2^ h β:2741	[62]
CS	Ag+ grafted MWNTs	Ben-C-Hex	20 °C, 50 wt.% benzene	J: 0.100 kg/m^2^ h β:4.5	At 1.5 wt.% loading: J: 0.357 kg/m^2^ h β:7.89	[63]
CS	S-CMS	Water-AC	50 °C, 5 wt.% water, 0.03 mbar	J: 1.10 kg/m^2^ h β:480	At 2 wt.% loading: J: 1.81 kg/m^2^ h β:832	[64]
CS	r-GO	Water-MeOH	30 °C, 10 wt.% water, 0.03 mbar	J: 0.230 kg/m^2^ h	At 1 wt.% loading: J: 340 kg/m^2^ h	[65]
CS/PVA	NH_2_- MWNTs	Water-IPA	25 °C, 30 wt.% water, 24 mbar	J: 1.80 kg/m^2^ h β:5	At 10 wt.% loading: J: 0.80 kg/m^2^ h β:99.5	[66]

**Table 3:** Examples of synthetic polymer-based mixed matrix membranes for ethanol dehydration.

Mixed Matrix Membrane	Filler Loading:	J (kg m^−2^ h^−1^)	Separation Factor β)	Reference
Cross-linked PVA-filled GO	1 wt.%	0.137	263	[10]
Polyimide-filled ZIF-8	12 wt.%	0.260	300	[67]
PVA-filled MWCNT	5 wt.%	0.080	500	[11]
Polyimide-filled MSS-1	12 wt.%	0.310	190	[67]
Cross-linked PVA-filled ZIF-8-NH_2_	7.5 wt.%	0.120	200	[68]

## Abbreviations

A_m_	Membrane area
β	separation factor
CNs	g-C_3_N_4_ nanosheets
CS	Chitosan
D	Diffusivity
EG	Ethylene glycol
GA	Glutaraldehyde
GO	Graphene oxide
J	Permeate flux
J_A_	Permeate flux of the compound A
K+MMT	Potassium montmorillonite
m_A_	Mass of the molecule A
MeOH	Methanol
MTBE	Methyl tert-butyl ether
MMM	Mixed matrix membrane
MOF	Metal-organic framework
PEG	Poly(ethylene glycol)
PHB	Polyhydroxybutyrate
PLA	Polylactic acid
POSS	Polyoctahedral oligomeric silsesquioxanes
PV	Pervaporation
PVA	Polyvinyl alcohol
PVMR	Pervaporation membrane reactor
PAN	Polyacrylonitrile
PVDF	Polyvinylidene fluoride
PDMS	Polydimethylsiloxane
POMS	Polyoctylmethyl siloxane
P	Permeability
S	Solubility
SA	Sodium alginate
SPVA	sulfonated polyvinyl alcohol
